# Minor Components Play an Important Role in Interspecific Recognition of Insects: A Basis to Pheromone Based Electronic Monitoring Tools for Rice Pests

**DOI:** 10.3390/insects9040192

**Published:** 2018-12-12

**Authors:** Qing-Hua Chen, Feng Zhu, Zhihua Tian, Wan-Min Zhang, Rong Guo, Wancai Liu, Lieming Pan, Yongjun Du

**Affiliations:** 1 Key Laboratory of Integrated Pest Management on Crops in Southwest, Institute of Plant Protection, Sichuan Academy of Agricultural Sciences, Ministry of Agriculture, Chengdu 610066, China; Fenglq2004@sina.cn; 2 Jiangsu Station of Plant Protection and Quarantine, Nanjing 210014, Jiangsu, China; zhufeng@jsagri.gov.cn (F.Z.); IPM@jsagri.gov.cn (Z.T.); 3 Liaoning Station of Plant Protection, Liaoning 110034, Shenyang, China; wmzh1@sina.com; 4 National Extension and Service Center of Agricultural Technology, Beijing 100125, China; guorong@agri.gov.cn (R.G.); liuwancai@agri.gov.cn (W.L.); 5 Department of Research and Development, NewCon Inc., Ningbo 315860, China; liemlngpan@sina.com; 6 Institute of Pesticide and Environmental Toxicology, Zhejiang University, Hangzhou 310058, China

**Keywords:** *Chilo suppressalis*, *Helicoverpa armigera*, *Scirpophaga incertulas*, *Mythimna separate*, sex pheromone, field trapping, interspecific recognition and reproductive isolation

## Abstract

Several lepidopteran species share the same pheromone blend consisting of (Z)-11-hexadecenal (Z11-16:Ald) and (Z)-9-hexadecenal (Z9-16:Ald) at different ratios and active doses. In rice pest *Chilo suppressalis*, (Z)-11-hexadecenol, (Z11-16:OH) and octadecanal (18:Ald) were identified as minor components in the pheromone gland of female moths, and these components were previously not considered as part of the sex pheromone of *C. suppressalis*. Z11-16:Ald, Z9-16:Ald and (Z)-13-octadecenal (Z13-18:Ald) frequently trapped other lepidopteran species, such as rice pests *Scirpophaga incertulas* and *Mythimna separate*, corn and vegetable pests *Helicoverpa armigera* in the field, suggesting a lack of specificity in the pheromone blend. Our data showed that the minor component Z11-16:OH did not have a synergistic effect on the attractiveness of the blend to *C. suppressalis*; however, pheromone mixtures containing Z11-16:OH failed in trapping male *H.* *armigera* moths. We confirmed the identity and specificity of the *C. suppressalis* sex pheromone and demonstrated that Z11-16:OH plays a key role in the reproductive isolation of *C. suppressalis*, *M. separata*, and *H.* *armigera* moths, and a similar role of Z9-18:Ald in that of *S. incertulas* and *C. suppressalis*. This phenomenon could be more widely applicable to interspecific interactions in the pheromone communication between insects, which is crucial to developing the electronic automatic counting device for automatically monitoring the pest population by pheromone trapping based on its species specificity.

## 1. Introduction

In the field, crowds of *C. suppressalis*, *S. incertulas*, *Mythimna separate*, and *H. armigera* moths occurred together, for instance, at the overlapping areas of rice, vegetables, and corn cropping, where the traps baited with the pheromone blends composed of *C. suppressalis* synthetic compounds would catch these species. Such false trapping has also been reported in other lepidopteran species, especially those that are closely related to each other [1,2,3,4,5,6]. Differences in diel rhythms of attraction and seasonal distribution were insufficient to maintain the reproductive isolation in these instances. The specificity of pheromone attraction is considered as the paramount premating reproductive isolating mechanism in moths among different species [3].

The components of the female sex pheromone of the rice pest *Chilo suppressalis* (Walker) (Lepidoptera: Pyralidae) were identified as three compounds containing (Z)-11-hexadecenal, (Z)-13-octadecenal, and (Z)-9-hexadecenal [7,8,9,10]. However, several other species share the same mixture of Z11-16:Ald and Z9-16:Ald at similar ratios, such as *Helicoverpa armigera* (Hübner) (Lepidoptera: Noctuidae) [11,12,13,14] and the rice pest *Scirpophaga incertulas* (Walker) (Lepidoptera: Crambidae) [10,15,16,17,18]. The ratios at which these two components remain active, ranged from 12:1 to 4:1 in *C. suppressalis* [19], from 4:1 to 2:1 in *S. incertulas* [10], and from 32:1 to 6:1 in *H. armigera* [20]. Z9-18:Ald was included in the synthetic pheromone blend of *S. incertulas* reported by Tatsuki [16], but not by others through the field evaluation of synthetic pheromone mixtures [10,17,21]. The sex pheromone of another migratory rice pest *Mythimna separata* (Walker) was identified to be (Z)-11-hexadecenal, hexadecanal, and (Z)-11-hexadecenol [22]. The general method of evaluation of the blend of insect sex pheromone is to simplify the composition of the mixtures by the pheromones attractiveness in the field. It has been reported that some minor components do not increase the attractiveness of the mixture, but play a role in the interspecific reproduction isolation [23,24]. Therefore, we must conclude that the current synthetic pheromone blends consisting of the three components do not reflect the complete blend for *C. suppressalis* and *S. incertulas*.

Knowing the specificity of pheromone blends is important for understanding the mechanisms of chemically-mediated mating behavior, as well as the interspecific chemical communication. Practically, a lack of specificity in synthetic pheromone blends means that non-target species caught in the pheromone traps will make it inaccurate to evaluate the pest population of the targeted species. Particularly, the electronic automatic counting system based on pheromone trapping works only when the system has caught the pure target insect species, which was determined by the specificity of the pheromone lures. In this study, we report and summarize our field evaluation work on the specificity of the *C. suppressalis* sex pheromone, and the mechanism of olfactory discrimination amongst the *C. suppressalis*, *S. incertulas*, *H. armigera* and *M. separate* from 2010–2015.

## 2. Materials and Methods

### 2.1. Insects

Rice plants, *Oryza sativa* L. (Poales: Poaceae), infested with *C. suppressalis* pupae were collected from Meishan, Sichuan (30°3′ N, 103°47′ E) in April of 2012 and transferred to a controlled environment room at 25 ± 1 °C and 75 ± 5% relative humidity under an ambient light: dark cycle (14 h:10 h). The rice plants were enclosed in a screen-covered metal frame. Newly emerged moths were collected and separated by sex twice a day, placed in screened cages (30 cm × 20 cm × 15 cm), and provided with a 10% sucrose solution soaked into cotton wool in a plastic dish prior to pheromone extraction.

### 2.2. Pheromone Extraction

Sex pheromone glands were removed from 3- to 5-day-old virgin female moths after approximately 6–7 h into the scotophase. The gland was everted by gentle pressure on the abdomen, gripped with the forceps and sliced from the abdomen with a razor blade such that the gland remained inflated. The glands were then dipped into a glass vial containing approximately 200 μL of redistilled hexane for 30 s. These extracts (8–10 glands per extract) were transferred into glass capillaries with pointed bases, concentrated under a slow stream of high purity nitrogen to about 20 μL, and stored at −20 °C for future analysis.

### 2.3. Pheromone Identification of C. suppressalis

The pheromone gland extracts of female moths were analyzed using Gas Chromatography coupled with Mass Spectrometry (GC-MS), and the identity of the main peaks was confirmed by GC peak enhancement using the synthetic reference compounds. Both the crude extract and the synthetic reference compounds were analyzed in an Agilent 6890GC-5975MS equipped with a DB-35 column (30 m × 0.25 mm × 25µm). The temperature was programmed at 60 °C for 1 min, followed by an increase of 10 °C per min until 180 °C was achieved, and then by an increase of 3 °C per min till 250 °C, which was maintained for 10 min afterwards. The temperature of the ion source was 230 °C, and the energy voltage 70 eV. Helium was used as the carrier gas.

### 2.4. Sources of Synthetic Reference Compounds

The pheromone components, Z11-16:Ald, Z9-16:Ald, Z13-18:Ald, Z11-16:OH, were purchased from Bedoukian Research Inc. (Danbury, CT, USA). Z9-18:Ald, 16:Ald, and 18:Ald were synthesized at our laboratory. Each compound was purified using silica gel flash chromatography, resulting in ≥97% purity (as confirmed by gas chromatography), then transferred to the clean glass vials and stored at −20 °C prior to use. Hexane (AR purity) was purchased from a local company and redistilled. Butylated hydroxytoluene (BHT) was manufactured by Nanjing Hualiming Chemical Inc. (Nanjing, China).

### 2.5. Field Trials

A series of field experiments were set up to test the relative efficacy of the synthetic pheromone lures containing different blends, ratios, and doses of pheromone components to attract the male *C. suppressalis*, *H. armigera*, *S. incertulas*, and *M. separata* moths. An experiment comparing the different ratios was then done at two locations, Ningbo (29°41′ N, 121°27′ E), Zhejiang (18 August–6 September 2010) and Meishan (30°04′ N, 103°50′ E), Sichuan (19 August—25 August 2010), to determine whether there were any geographical differences in the responses of *C. suppressalis* males to the different pheromone ratios. A third experiment on the effect of the pheromone dose (only for the ratio of 490:49:60 for Z11-16:Ald:Z9-16:Ald:Z13-18:Ald) on trap catches of male *C. suppressalis* was done in Ningbo, Zhejiang between 5 May–21 May 2010. During the experiment in the rice fields in Ningbo from 8 August to 20 September 2011, we compared the *C. suppressalis* of male moths’ in trap catches baited with single components and different ratios of the four previously identified components of the *C. suppressalis* sex pheromone, Z11-16:Ald:Z9-16:Ald:16:Ald:Z13-18:Ald. A fourth experiment to evaluate the effect of more complex blends of pheromone components, including minor components, on the trap catches of male *C. suppressalis* was done at three locations, Ningbo, Zhejiang (28 June–30 July 2012), Meishan, Sichuan (30 June–25 July 2012), and Shaoyang (27°22′ N, 111°5′ E), Hunan (12 August–30 August 2012). In a fifth experiment, the effect of the *S. incertulas* pheromone component Z9-18:Ald on the attractiveness of blends was evaluated in the rice field in Ningbo, Zhejiang (3 June–29 June 2013). In all the treatments, the ratio of Z11-16:Ald, Z9-16:Ald, Z13-18:Ald remained the same at 490:49:60, but the ratios and quantities of the minor components, 16:Ald, Z11-16OH and 18:Ald were varied. In the final experiment in cotton fields in Datukou (30°48′ N, 117°05′ E), Anhui (10 September–22 September 2012), these complex blends were evaluated to determine whether they also captured *H. armigera* males. The trapping experiments for *Myhtimna separata* were carried out in Ningbo (29°41′ N, 121°27′ E), Zhejiang, and Yuxi (24°35′ N, 102°52′ E), Yunnan.

All the pheromone dispensers or lures were made from PVC capillary tubing (ca. 80 mm length, inner diameter 0.6 mm, and outer diameter 1.1 mm) (NewCon Inc., Ningbo, China). Pheromone components for experimentation were mixed, followed by the adding of corn oil as the solvent to achieve the desired concentrations. Each solution was stirred using magnetic stirrers (XK95-B, Jiangyan, Jiangsu, China), and then 10 μL of solution injected into PVC capillary tubing dispensers at the required doses. Both ends of the dispensers were heat sealed at about 150 °C in 0.2–3 s (FRE-450BX1, Jiangnan, Wenzhou, China). The dispensers were shipped by courier to the test locations in sealed aluminum foil bags and stored at −20 °C for future use. In the experiments in the rice paddy fields, the traps baited with the pheromone blends were normally hung about 10 cm below the height of the rice plants, whilst in the experiments in the cotton fields, the traps were placed 1 m high above the ground. The flying moth traps (FMT) that were used were purchased from NewCon Inc. (Ningbo, China), and there were either five or six replicate traps per treatment used for all the experiments. The FMT trap was assembled with an inverted plastic mesh funnel, a plastic busket and a pheromone lure rod. We set the control based on the experimental objectives. No pheromone lure was installed in the trap in the control. The distance between traps was 30 m.

### 2.6. Data Analysis

Data were analyzed by two-way ANOVA with treatments and replicates as variables in the software package SAS 9.2 (SAS Institute Inc., Cary, NC, USA). Duncans multiple-range tests were used for multiple comparisons between means.

## 3. Results

### 3.1. Analysis of the Pheromone Extract Using GC-MS

**Figure 1:**
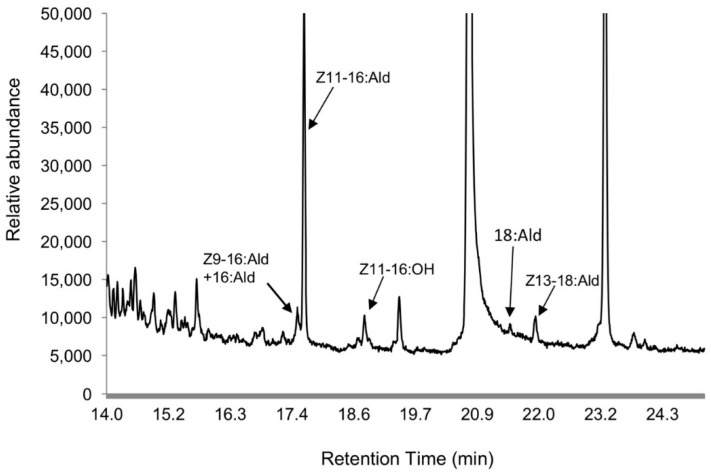
Gas Chromatography coupled with Mass Spectrometry (GC-MS) analysis of crude pheromone extracts from female *Chilo suppressalis* moths.

The major component in the crude pheromone extracts was Z11-16:Ald. Z9-16:Ald, 16:Ald, Z13-18:Ald, and Z11-16:OH were also detectable, but only in small amounts as minor components. 18:Ald was a very minor component and detectable only in a few extracts (Figure 1). The largest peaks appeared to be Z11-hexadecenoic acid and dibutyl phthalate based on the mass spectra, which were non-pheromone compounds. The average ratio of Z11-16:Ald, Z9-16:Ald + 16:Ald and Z13-18:Ald was 100:29.8 ± 21.4:8.8 ± 1.9 from five extracts.

### 3.2. Attractiveness of Synthetic Pheromone Mixtures to male C. suppressalis Moths in the Field

**Figure 2:**
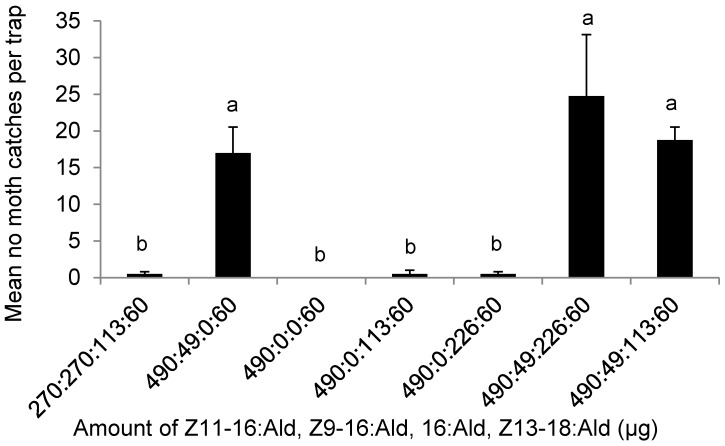
The average number of male *Chilo suppressalis* caught in the rice fields in Ningbo between 8 August and 20 September 2011 per trap per night, by lures made up of single and mixed blends of the pheromone components Z11-16:Ald, Z9-16:Ald, 16:Ald, and Z13-18:Ald, in the ratios indicated along the *x* axis. Each treatment was replicated five times. Treatment bars represent the standard errors; bars with different letters above them are significantly different from each other in the number of male moths captured (*p* < 0.05).

In the first experiment, pheromone mixtures without Z9-16:Ald caught significantly fewer *C. suppressalis* moths than any other treatment (DF = 8, F = 5.05, *p* = 0.0035) (Figure 2). The ratio of Z11-16:Ald to Z9-16:Ald was the key factor influencing the number of moths trapped; Z11-16:Ald and Z9-16:Ald in a ratio of 490:49 captured significantly more moths than any other treatment (DF = 9, F = 5.96, *p* = 0.0007) (Figure 2). Furthermore, the addition of 16:Ald in the mixture appeared to not increase the moth catches in Ningbo (DF = 5, F = 1.6, *p* = 0.29) (Figure 2).

**Figure 3:**
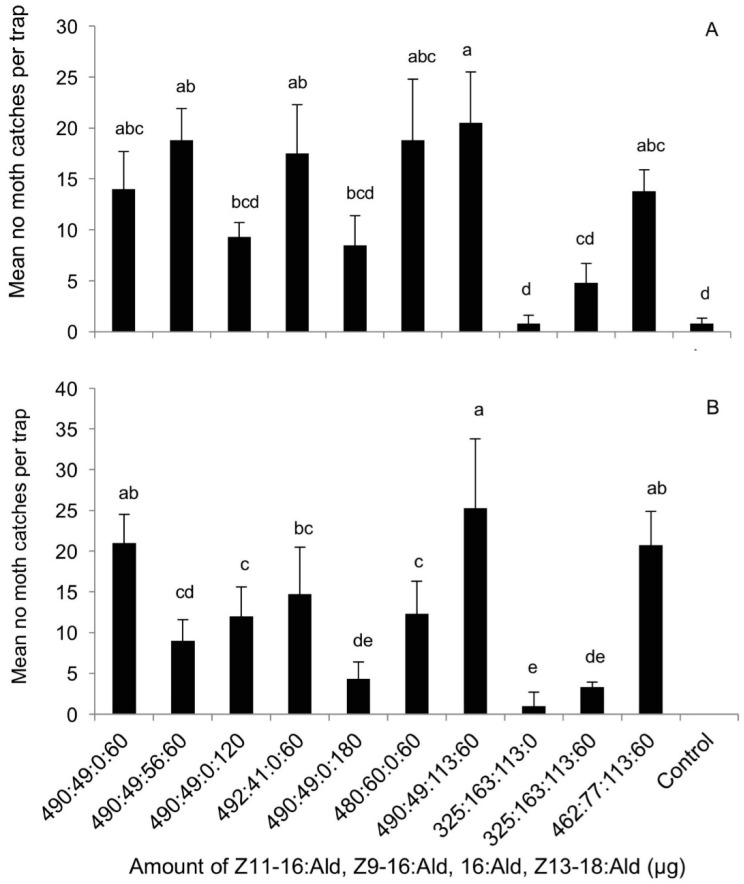
The average number of male *Chilo suppressalis* caught per trap per night in the rice fields in (**A**) Ningbo, Zhejiang (18 August–6 September 2010) and (**B**) Meishan, Sichuan (19 August–25 August 2010), by lures made up of single and mixed blends of the pheromone components Z11-16:Ald:Z9-16:Ald:16:Ald:Z13-18:Ald, in the ratios indicated along the *x* axis. Each treatment was replicated five times. Treatment bars represent the standard errors; the bars with different letters above them are significantly different from each other in the number of male moths captured (*p* < 0.05).

In the second experiment, trap catches at both field sites (Ningbo and Meishan) demonstrated that the complete blends of 490:49:113:60 blend of Z11-16:Ald, Z9-16:Ald, 16:Ald, and Z13-18:Ald could capture significantly more *C. suppressalis* moths than any other mixtures missing one of the components (Ningbo test: DF = 14, F = 5.01, *p* < 0.0001; Meishan test: DF = 11, F = 9.82, *p* < 0.0001) (Figure 3A,B). The importance of Z13-18:Ald in the pheromone blends was also clear: when this component was missing, the lures were no longer attractive, so that significantly fewer moths would be captured than any other pheromone treatments. The number was not significantly different from the control (Meishan: DF = 4, F = 9.56, *p* = 0.025; Ningbo: DF = 5, F = 0.96, *p* = 0.5) (Figure 3A,B). Moreover, the experiment also showed that the optimal amount of Z13-18:Ald in the mixture was 60 µg per lure, but the attractiveness of the mixture decreased when the amount of this component increased. The role of 16:Ald was different for the two locations. It affected the attractiveness of the pheromone mixture in Meishan but not in Ningbo.

**Figure 4:**
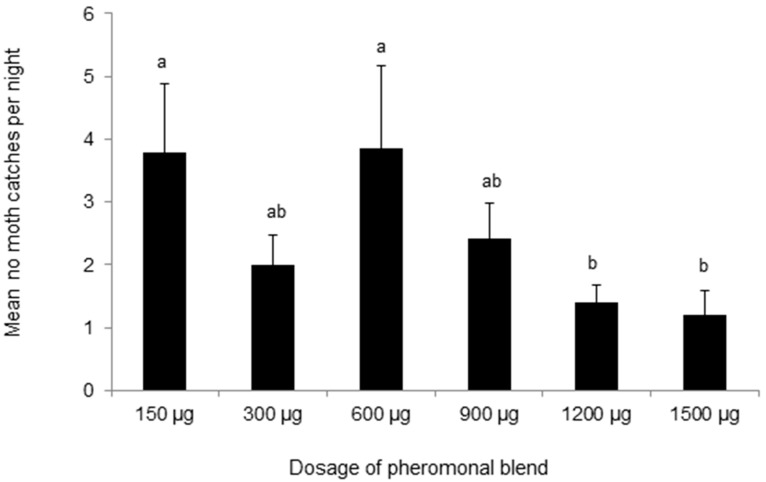
Dose-response of *Chilo suppressalis* to synthetic pheromone blends in Ningbo, Zhejiang (5 May–21 May 2010). The treatment bars represent the standard errors; the bars with different letters above them are significantly different from each other in the number of male moths captured (*p* < 0.05). The blend ratio of Z11-16:Ald:Z9-16:Ald: Z13-18:Ald was 490:49:60 in all lures.

The third experiment that evaluated the effect of different doses of the blends in a ratio of 490:49:60 of Z11-16:Ald, Z9-16:Ald, Z13-18:Ald on trap catches demonstrated that the blends containing a total of 600 μg and 150 μg per lure would catch the highest number of moths. Larger amounts of this blend would decrease in overall attractiveness (DF = 10, F = 2.74, *p* = 0.02) (Figure 4).

**Figure 5:**
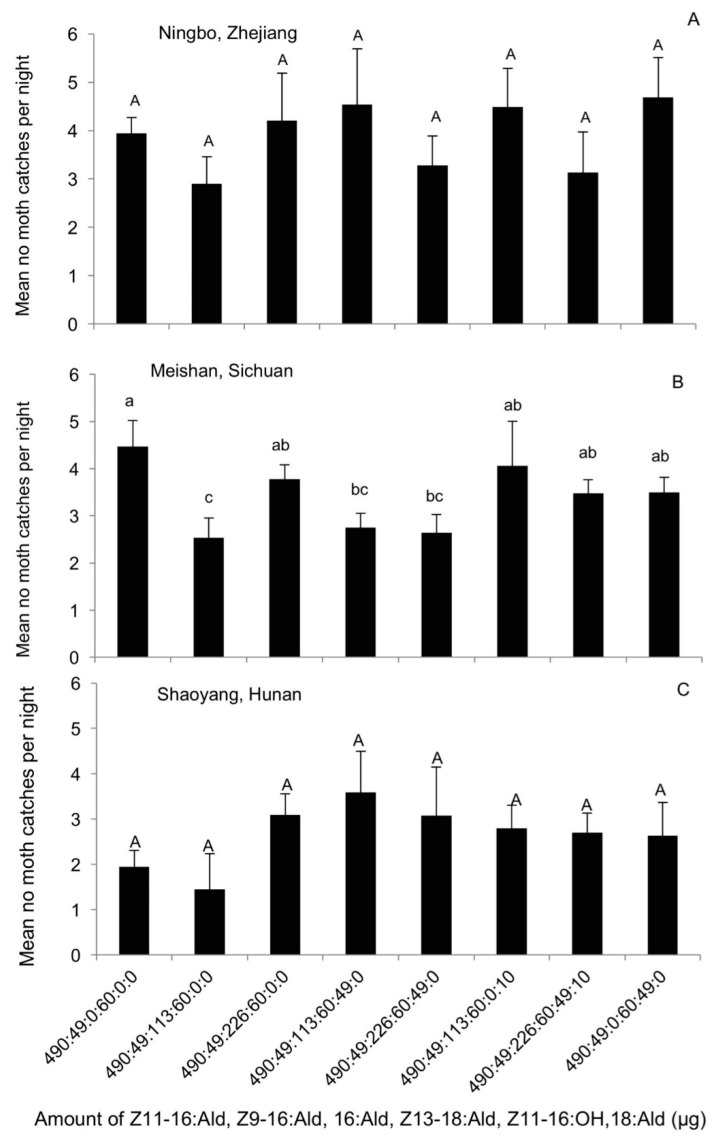
The average number of male *Chilo suppressalis* moths caught per trap per night in the rice fields in (**A**) Ningbo, Zhejiang (28 June–30 July 2012); (**B**) Meishan, Sichuan (30 June–25 July 2012); (**C**) Shaoyang, Hunan (12 August–30 August 2012), by lures made up of mixed blends of the pheromone components Z11-16:Ald, Z9-16:Ald, Z13-18:Ald, 16:Ald, Z11-16:OH, 18:Ald, in the ratios indicated along the *x* axis. Each treatment was replicated six times. Treatment bars represent the standard errors; bars with different letters above them are significantly different from each other in the number of male moths captured (*p* < 0.05).

In the fourth experiment, the data suggested little geographic variation in the attractiveness of each mixture (Figure 5A–C). There was no statistically significant difference in the trap catches between the different pheromone treatments in Ningbo (DF = 7, F = 1.41, *p* = 0.23) or in Shaoyang (DF = 10, F = 1.33, *p* = 0.28). Amounts of Z11-16:OH and 18:Ald had no significant effect on moth catches. In Meishan, the effect of 16:Ald was difficult to interpret, but the overall difference in the number of moth catches was small (DF = 12, F = 2.6, *p* = 0.013).

**Figure 6:**
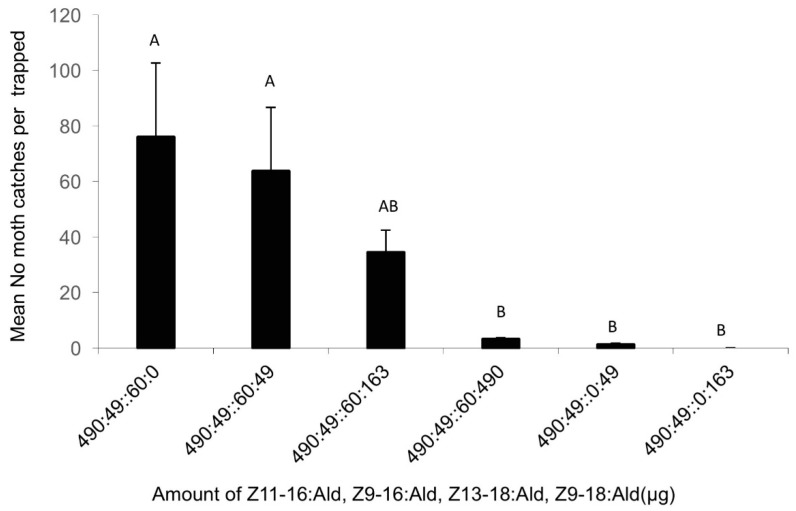
The average number of male *Chilo suppressalis* moths caught per trap per night in the rice fields in Ningbo, Zhejiang (3 June–29 June 2013), by lures made up of mixed blends of the pheromone components Z11-16:Ald, Z9-16:Ald, Z13-18:Ald, Z9-18:Ald, in the ratios indicated along the *x* axis. Each treatment was replicated five times. Treatment bars represent the standard errors; bars with different letters above them are significantly different from each other in the number of male moths captured (*p* < 0.05).

In the fifth experiment, the data showed that the blend containing Z9-18:Ald, one of the sex pheromone components of *S. incertulas*, attracted less or even no *C. suppressalis* moths (Figure 6). While the amount of Z9-18:Ald increased in the blend, fewer moths were trapped. Nothing but very few *C. suppressalis* was caught when the amount of Z9-18:Ald was higher than 490 μg in the mixture of each pheromone lure (DF = 9, F = 3.1, *p* = 0.018).

### 3.3. Attractiveness of Synthetic Pheromone Mixtures to H. armigera in the Field

**Figure 7:**
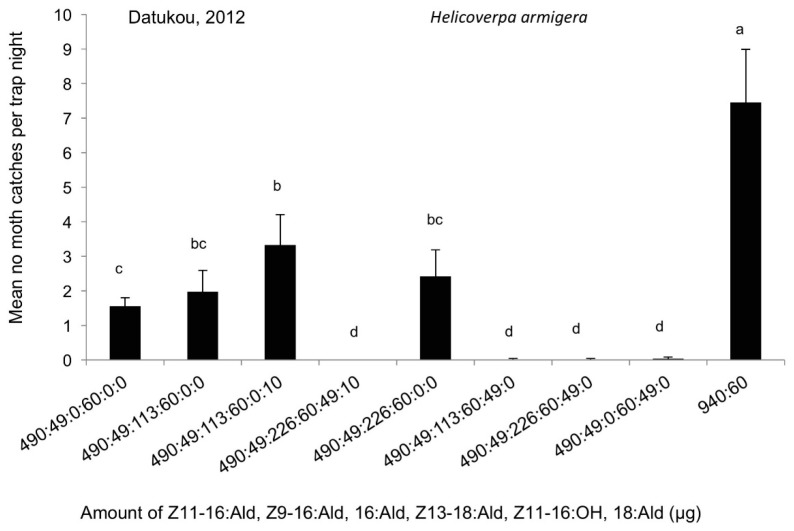
The average number of male *Helicoverpa armigera* caught per trap per night in the cotton fields in Datukou, Anhui (10 September–22 September 2012), by lures made up of mixed blends of the pheromone components Z11-16:Ald, Z9-16:Ald, Z13-18:Ald, 16:Ald, Z11-16:OH, 18:Ald, in the ratios indicated along the *x* axis. Each treatment was replicated six times. Treatment bars represent the standard errors; bars with different letters above them are significantly different from each other in the number of male moths captured (*p* < 0.05).

The reference pheromone blend consisting of Z11-16:Ald, Z9-16:Ald, and Z13-18:Ald (490 µg:49 µg:60 µg) caught a small number of *H. armigera* male moths in the field (Figure 7), although not as many as the blending of Z11-16:Ald and Z9-16:Ald (940:60 µg), widely used in China for monitoring *H. armigera* populations. In fact, the blend of Z11-16:Ald and Z9-16:Ald (940:60 µg) at a 1 mg dosage captured significantly more *H. armigera* moths than any other treatments (DF = 13, F = 7.36, *p* < 0.0001). The addition of 16:Ald generally increased the number of moth catches, whilst the addition of 16:Ald and 18:Ald did not significantly increase the attractiveness of the reference pheromone blend (DF = 8, F = 0.38, *p* = 0.9). In contrast, the addition of Z11-16:OH completely suppressed the attractiveness of the reference pheromone blend to *H. armigera*; significantly fewer moths were captured when Z11-16:OH was added to the blend of Z11-16:Ald, Z9-16:Ald, and Z13-18:Ald (490 µg:49 µg:60 µg) than in any other treatments (DF = 12, F = 4.02, *p* = 0.0006).

### 3.4. Attractiveness of Synthetic Pheromone Mixtures to M. separata in the Field

**Figure 8:**
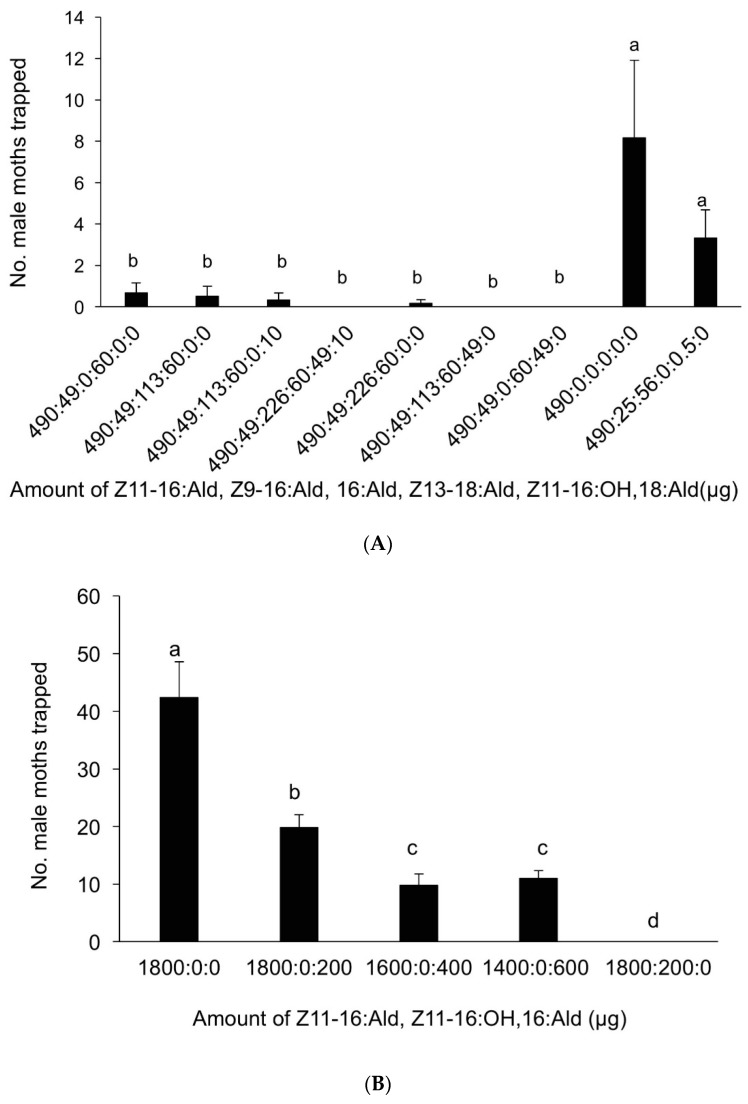
(**A**) The average number of male *Myhtimna separata* caught per trap in the rice fields in Ningbo, Zhejiang (10 September–22 September 2012), by lures made up of mixed blends of the pheromone components Z11-16:Ald, Z9-16:Ald, Z13-18:Ald, 16:Ald, Z11-16:OH, 18:Ald, in the ratios indicated along the *x* axis. Each treatment was replicated six times. Treatment bars represent the standard errors; bars with different letters above them are significantly different from each other in the number of male moths captured (*p* < 0.05). (**B**) The average number of male *Mythimna separata* caught per trap in the rice fields in Yuxi, Yunnan (10 June–22 June 2015), by lures made up of mixed blends of the pheromone components Z11-16:Ald, 16:Ald, and Z11-16:OH, in the ratios indicated along the *x* axis. Each treatment was replicated six times. Treatment bars represent the standard errors; bars with different letters above them are significantly different from each other in the number of male moths captured (*p* < 0.05).

The reference pheromone blend consisting of Z11-16:Ald, Z9-16:Ald, and Z13-18:Ald (490 µg:49 µg:60 µg) caught a small number of *M. separata* male moths in the field (Figure 8A), although fewer moths were trapped by the blend of Z11-16:Ald and 16:Ald, used widely in China (Figure 8B) for monitoring *M. separata* populations. In fact, Z11-16:Ald captured significantly more moths than any other treatments. The addition of 16:Ald actually decreased the number of moth catches (DF = 59, F = 9.558, *p* < 0.001). Moreover, the addition of Z11-16:OH completely suppressed the attractiveness of the reference pheromone blend to the *M. separata* (Figure 8B) (DF = 39, F = 28.029, *p* < 0.001).

## 4. Discussion

In nature, the olfactory systems of insects are so selective that, for example, male moths discriminate the sex pheromones produced by the conspecific female moths from the similar compounds or mixtures of compounds that have only minimal differences in the structure (e.g., chirality), ratio or dose within the mixture. The overall specificity of any sex pheromone is determined by the complete blend of compounds released by conspecific insects, including dominant and minor components. In response to a complete blend, compared with a partial blend, the time spent on activation and landing of orientation flights in moths is decreased; and the proportion of males initiating take-off, lock-on, close-in, and touchdown phases of upwind flight is increased [25]. The blend consisting of Z11-16:Ald, Z9-16:Ald, and Z13-18:Ald is simplified to be the complete pheromone blend for *C. suppressalis* [8,10,19]. Furthermore, the ratio of Z11-16:Ald, Z9-16:Ald, and Z13-18:Ald within the complete blend plays a key role in its attractiveness to male *C. suppressalis* moths. Our field data from three locations showed little geographic variation in responses, where 16:ald slightly affected the responses of mixtures in Meishan, Sichuan. However, our data also showed that changes in the major component (three components) ratios within the blend failed to stop other species, such as *H. armigera*, *M. separata*, and *S. incertulas*, from being trapped. It has shown that a single molecular determinant can function as a behavior modulator, as well as an all-or-nothing initiator of a complex species-specific behavioral sequence [26], which can result in no effect of the blend of major compounds on specificity; and this may account for our observation on *H. armigera*, *M. separata*, and *S. incertulas*. The responsive range of blend ratios, Z11-16:Ald and Z9-16:Ald, among *C. suppressalis*, *S. incertulas*, and *H. armigera* was clearly overlapping.

Minor components are generally considered to be very important in modulating the attractiveness of pheromone mixtures, for example, Z9-16:Ald and Z13-18:Ald in *C. suppressalis* here, and E10E12-16:OH and E10-16:Ald in *Maruca vitrata* (F.) (Lepidoptera: Crambidae) [27]. In sympatric lepidopteran species on maize plants, the addition of Z11-16:Ald, a compound of the *Sesamia nonagrioides* (Lef.) (Lepidoptera: Noctuidae) pheromone, to Z11-16: OAc, significantly reduced the attraction of *Mythimna unipuncta* (Haworth) (Lepidoptera: Noctuidae) males both in the wind tunnel and in the field, as well as the number of sympatric clover cutworm, *Discestra trifolii* Walker (Lepidoptera: Noctuidae) under the field conditions. The addition of Z9-16: OAc, a minor component of the *M. unipuncta* pheromone blend, reduced the number of *S. nonagrioides* captured in the field traps that were baited with the *S. nonagrioides* lure, which showed the significance of such inhibition in the reproductive isolation of sympatric species that attack maize [28]. In three species of the genus *Adoxophyes*, the ratios and doses of Z9-14:OAc and Z11-14:OAc, as the major components, preferentially attracted different species; however, minor components, in addition to the relative ratios of the two major components, played an important role in reproductive isolation between *Adoxophyes* spp. in southern and midwestern Korea, where these species occurred sympatrically [29]. Z6-12:OAc added at the rate of 0.1% or more to the known attractant blend increased the percentage of *Grapholita molesta* (Busck) (Lepidoptera: Torticidae) to 92% of all species within the wider group of *Grapholita* (=*Cydia*) spp., compared with 67% without this compound [30]. Such interactions have also been seen in *Synanthedon tipuliformis* (Clerck) (Lepidoptera: Sesiidae) [31].

It has been proposed that multicomponent pheromones consist of primary components responsible for eliciting the long-range (>1 m) upwind orientation and the secondary components, which, in combination with primary components, elicit the close-range behaviors such as landing and copulation [32]. This was supported by studies on *G. molesta* [33], *Argyrotaenia velutinana* [34], *Trichoplusia ni* [35,36], and *Panolis flammea* [37]. However, the minor components also function to enhance the male sensitivity to the pheromone and the specificity of the signal. It is suggested that the ‘active space’ of the pheromone is a function of the upper and lower concentration thresholds for the entire blend of components, instead of simply for the major component [38]. The ‘active space’ was previously defined as the space where the major pheromone component was within both the lower and upper thresholds for a particular behavior [39]. In a blend ratio approximating to that emitted by *G. molesta* females, the three components (Z8-12:OAc, E8-12:OAc and Z8-12:OH) elicited the increases in both long-range and close-range behavior in the male response sequence. Hence, these components acted as a unit. An additional fourth component, 12:OH, had small but significant effects on the hair-pencil display when Z8-12:OH was at suboptimal levels, in contrast to the stronger behavioral effects previously ascribed to 12:OH. Z8-12:OH appeared to be important to reproductive isolation between *G. molesta* and *G. prunivora* (Walsh) (Lepidoptera: Tortricidae), because this component virtually eliminated the capture at 10%. There was a strong correlation between the pheromone plume behaviors of pre-flight wing fanning in the course of walking and upwind flight, suggesting that these behaviors may be closely associated, functioning to locate the pheromone source by ground or air, respectively [40]. In the behavioral tests, body extracts of females elicited close-range attraction and wing-fanning responses only by conspecific, but not by heterospecific males, supporting the hypothesis of the close-range species-specific sex pheromone blends [41].

Three American heliothine species have been studied, *Helicoverpa zea* (Boddie) (Lepidoptera: Noctuidae), *Helicoverpa virescens* F., and *Helicoverpa subflexa* [42]. *Helicoverpa zea* and *H. virescens* released the same major component Z11-16:Ald, and the different combination of trace components were essential to the pheromonal activities and the specificity of chemical signals [43]. The effects of the different pheromone components of *S. nonagrioides*, *M. unipuncta,* and *Ostrinia nubilalis* (Hübner) (Lepidoptera: Crambidae) on the behavior of *H. armigera* males in the wind tunnel were reported [44,45]. Z11-16:OH appeared to act antagonistically, along with another compound Z11-16:Ac, when it was added to the *H. zea* pheromone blend [46].The electrophysiological research showed that there were separate populations of receptors located on the antennae of the males of *H. zea*. The more sensitive population of receptors was selective for Z11-16:Ald, a conspecific component, and Z9-14:Ald, whilst the less sensitive population responded to Z9-14:Ald, a component found in the pheromone blend of a sympatric species, *H. virescens* that inhibited the attraction of males of *H. zea* and provided a physiological basis on which *H. zea* males could distinguish the interspecific repellent from the conspecific pheromone blend. It is likely that this discrimination has contributed to the reproductive isolation between the two species [47]. This specificity may be brought about through a number of mechanisms: (1) The use of additional pheromone components [23,48], (2) The utilization of unique and precise ratios of the shared components in conjunction with a comparably precise male response, and (3) The temporal differences in diel periodicities of sexual activity [6], or (4) Female-induced plant odors in combination with the sex pheromone [49].

**Figure 9:**
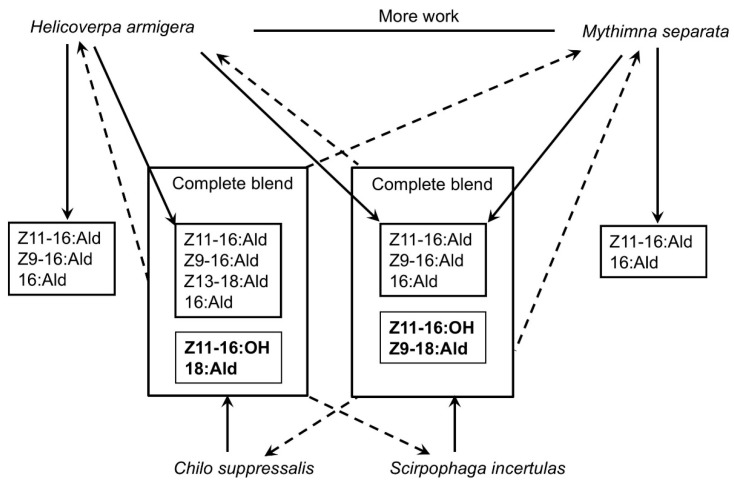
The diagram of the relationship between minor components and species specificity. Solid arrow means attractiveness. Dotted arrow means inhibition of minor components for species specificity.

The mixture designed in the field tests described here clearly showed that the three-component blend was able to attract a significant number of *H. armigera* and *M. separata* moths in the field, which was not acceptable in monitoring populations using the pheromone trap, especially in the electronic automatic counting monitoring system. The addition of Z11-16:OH completely suppressed the attractiveness of blends to *H. armigera*, *M. separata*, and *S. incertulas* moths. 16:OH and Z11-16:OH could also act as the attraction inhibitors when added to the blend of the *H. armigera* pheromone [20,45,50,51]. They interfered with the attraction of heterospecific males [52]. In most field tests published, only the simplest mixture of compounds active in the EAG were formulated, and the focus was on the moth catches of targeted insects. In *C. suppressalis*, the addition of Z11-16:OH did not increase or decrease the moth catches of *C. suppressalis* by the pheromone blends evaluated, but this compound played a key role in the specificity of the pheromone blends between species *C. suppressalis* and *H.armigera*, thereby separating the sympatric species. A similar role of Z9-18:Ald was confirmed for the specificity of pheromone blends in separating the species of *C. suppressalis* and *S. incertulas*. Nevertheless, whether it functions in the long distance or in the short distance needs be clarified by further behavioral experiments. Furthermore, for practical purposes in accurate population monitoring, especially for implementing the electronic automatic counting devices for automatically monitoring the pest population by pheromone trapping, Z11-16:OH and 18:Ald should be included in the synthetic pheromone mixture of *C. suppressalis* to represent a complete pheromone blend, and Z9-18 should also be included in *S. incertulas* (Figure 9).

## 5. Conclusions

In conclusion, we demonstrated the important role of minor components in the species specificity of insect sex pheromones. It has proved that Z11-16: OH in plays a key role in the reproductive isolation of *C. suppressalis*, *M. Separata* and *H. armigera*, and the role of 16:Ald varied by the geographical region. Z9-18: Ald plays a similar role in *S. incertulas* and *C. suppressalis*. Therefore, the whole pheromone composition of *C. suppressalis* should include Z11-16:OH, 18:Ald and 16:Ald. Sex pheromone of *S. incertulas* should add Z11-16: OH and Z9-18:Ald.
